# Laparoscopic Repair of a Type IV Hiatal Hernia Using Gore Bio-A® Mesh: A Case Report and Literature Review

**DOI:** 10.7759/cureus.87391

**Published:** 2025-07-06

**Authors:** Luis E Ocampo-Guzmán, Braulio A Crisanto-Campos, Emmanuel M DelCampo-Madariaga, Karla M Biviano-Andrade

**Affiliations:** 1 General Surgery, Hospital Regional Universitario de Colima, Colima, MEX; 2 General Surgery, Unidad de Cirugía y Endoscopia Gastrointestinal Mexicana, Mexico City, MEX

**Keywords:** absorbable mesh, anti-reflux surgery, hiatal hernia repair, laparoscopic nissen’s fundoplication, type iv hiatal hernia

## Abstract

Type IV hiatal hernia represents a complex and rare form of hiatal hernia, characterized by the protrusion of multiple abdominal organs, such as the stomach, spleen, and colon, into the mediastinum. The surgical approach is complex and requires meticulous technique to reduce the risk of complications and recurrences. We report the case of a patient with a type IV hiatal hernia who underwent laparoscopic repair with crural plasty and placement of Gore Bio-A® absorbable mesh (W. L. Gore & Associates, Newark, DE, USA), anti-reflux surgery (Nissen fundoplication), and simultaneous cholecystectomy. Postoperative evolution was satisfactory, with a one-year follow-up period showing no recurrences or complications. The specific surgical indications for type IV hernias are discussed, along with the current evidence on the use of absorbable meshes in hiatal repair.

## Introduction

Hiatal hernia is classified as an acquired diaphragmatic defect and can be categorized into four types, with type I being the most frequent (approximately 90%). Type IV hernias constitute the most advanced and rare form, with herniation of intra-abdominal organs other than the stomach, such as the spleen, colon, or small intestine [1,2]. These hernias present an increased risk of serious complications, including strangulation, necrosis, and obstructive symptoms [1,3].

Surgical management of type IV hiatal hernias should be timely and technically adequate. Laparoscopic surgery has gained preference over the open approach because of its advantages in recovery and morbidity [1,4,5]. Nevertheless, the wide hiatal defect in these cases increases the probability of recurrences, so the use of mesh for repair has been increasingly implemented [6,7].

Absorbable meshes, such as Gore Bio-A® (W. L. Gore & Associates, Newark, DE, USA), are composed of a three-dimensional matrix of polyglycolic acid and trimethylene carbonate, which promotes tissue integration and reduces complications associated with permanent meshes, including erosion or excessive fibrosis [6,8,9]. However, clinical evidence on its medium- (6 to 12 months) and long-term (>12 months) efficacy is still under development and is an active field of research [6,10-12].

## Case presentation

A 55-year-old female patient with a history of biliary colic and abdominal pain was consulted for recurrent episodes of pain in the right hypochondrium. During the preoperative evaluation for elective cholecystectomy, a chest X-ray was performed, showing the presence of the stomach in the mediastinum, which prompted further studies. Esophageal manometry and pH monitoring were not performed, as the patient had no dysphagia or significant reflux symptoms that would have indicated esophageal motility disorders.

**Figure 1:**
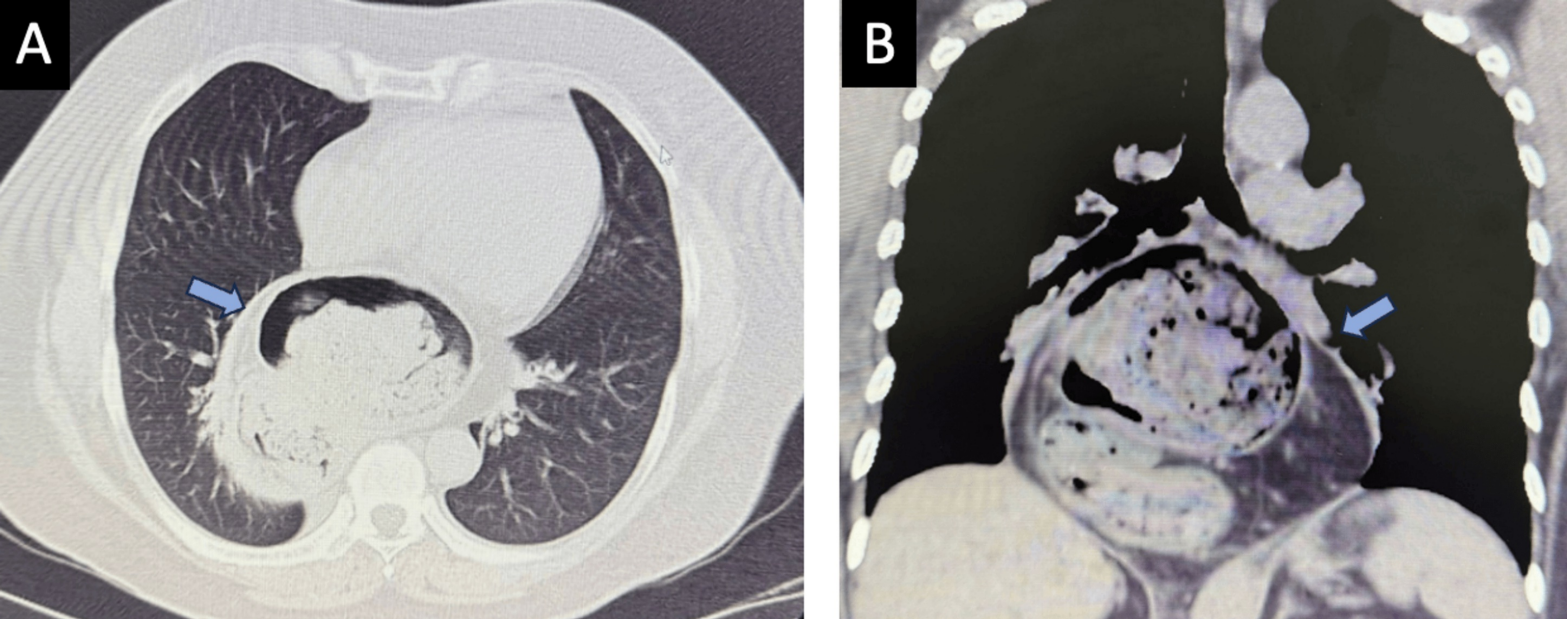
Thoracoabdominal computed tomography (A-B) Type IV hiatal hernia involves the gastric fundus, a portion of the splenic flexure of the colon, and the upper pole of the spleen.

The thoracoabdominal computed tomography and upper endoscopy showed a type IV hiatal hernia, with herniation of the gastric fundus, spleen, and a portion of the splenic angle of the colon into the mediastinum (Figure 1A-1B).

**Figure 2:**
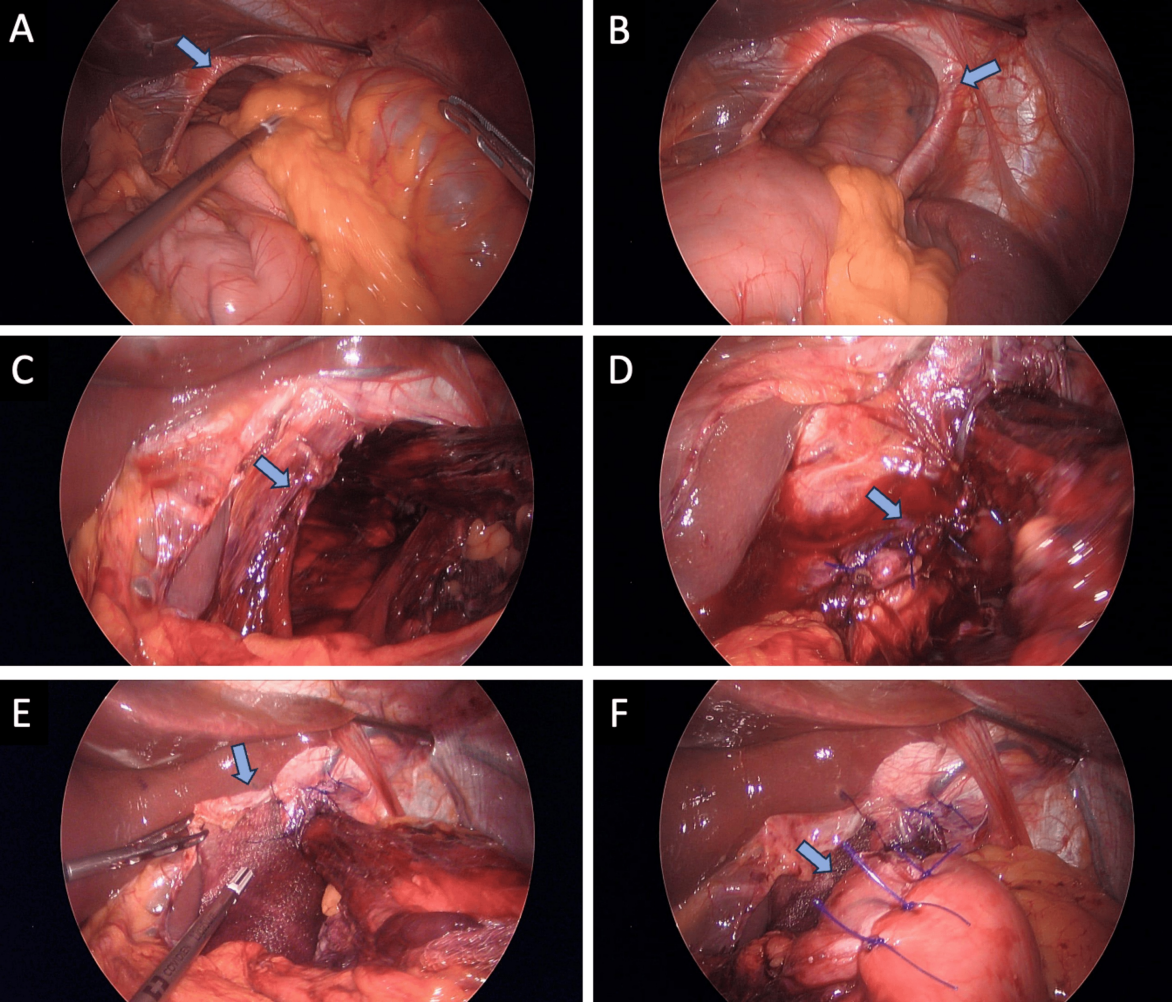
Laparoscopic surgery (A-B) A hernial defect is observed with a reduction of the splenic angle of the colon. (C-D) Closure of the crural defect is performed with a nonabsorbable suture. (E) Placement of absorbable mesh Gore Bio-A®. (F) Nissen-type fundoplication.

Elective laparoscopic surgery was scheduled. In the operating room, careful dissection of the esophagus and reduction of the herniated organs were performed, preserving their vascularization. Crural closure was performed with a non-absorbable suture and reinforcement with absorbable Gore Bio-A® mesh. Subsequently, Nissen fundoplication was used to control gastroesophageal reflux, followed by elective cholecystectomy without complications (Figure 2A-2F).

**Figure 3:**
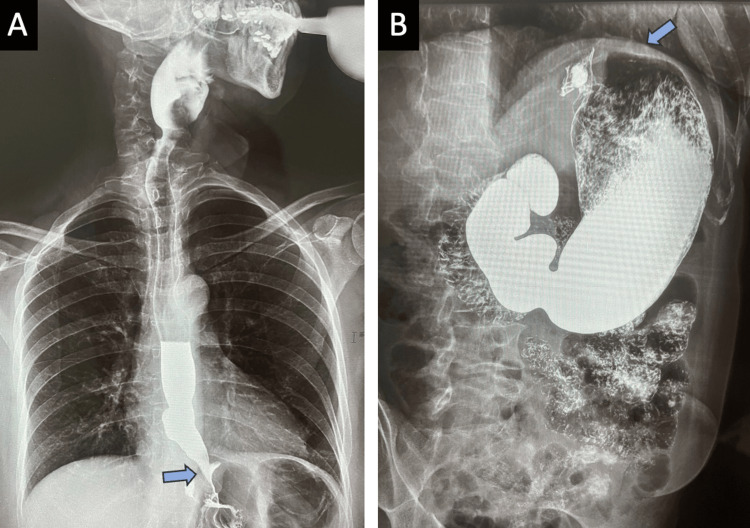
Esophagogram No recurrence of the hernial defect was observed.

The patient experienced a favorable immediate postoperative period, characterized by rapid recovery, tolerance to the oral route, and early mobilization. At 12-month follow-up, she was found to be asymptomatic, with no clinical or imaging evidence of recurrence or complications related to the intervention (Figure 3A-3B).

## Discussion

Type IV hiatal hernia requires surgical attention due to the high risk of severe complications, such as strangulation, organ necrosis, and obstructive symptoms [1-3]. The laparoscopic approach is currently the gold standard, offering less postoperative pain, shorter hospital stays, and accelerated functional recovery compared to open surgery [1,4,5].

A significant challenge in repair is the high recurrence rate when the hiatal defect is extensive, which is often the case in type IV hernias. The use of mesh to reinforce hiatal repair has been shown to reduce recurrence [6,12]. However, permanent meshes have been associated with complications such as esophageal erosion, fibrosis, or chronic pain [4].

Absorbable meshes, such as Gore Bio-A®, have emerged to promote a favorable balance, which is absorbed and replaced by functional scar tissue within six months, thereby decreasing long-term complications [6,8,9,11]. Recent studies report recurrence rates around 9-15% with this type of mesh, with a low incidence of complications [6,8,10]. Compared to primary suture repair, which is associated with recurrence rates up to 42%, mesh reinforcement significantly reduces recurrence risk. Permanent mesh repairs exhibit lower recurrence rates (10-15%) but have been associated with complications, including erosion and chronic pain [6,8,10,12].

In our case, the Gore Bio-A® repair was successful, with no recurrences or complications reported over the year. Nissen fundoplication added reflux control, an essential component in the treatment of hiatal hernias [1]. Concomitant cholecystectomy was performed to avoid future interventions and minimize risks, a strategy recommended in patients with associated cholelithiasis [13].

It is essential to individualize the use of mesh in each patient, considering factors such as the size of the defect, tissue quality, and the risk of infection [6,12,14]. More prospective and long-term studies are required to consolidate the role of absorbable meshes in this pathology.

## Conclusions

Laparoscopic repair of type IV hiatal hernias represents the current therapeutic standard, with clear advantages over open surgery. The use of absorbable mesh is a valuable tool for reinforcing the repair, reducing the recurrence rate, and offering a favorable safety profile. The surgical approach should be comprehensive, contemplating reflux control. Prolonged follow-up is recommended to evaluate long-term results.
